# Clinical Outcomes of Anorganic Bone Matrix Combined With a P-15 Osteogenic Cell-Binding Peptide (ABM/P-15) in One- and Two-Level Transforaminal Lumbar Interbody Fusion (TLIF): A Propensity-Matched Comparison

**DOI:** 10.7759/cureus.100339

**Published:** 2025-12-29

**Authors:** Mitsuhiro Nishizawa, Steven D Glassman, Mladen Djurasovic, Charles H Crawford, Jeffrey Gum, John R Dimar, R. Kirk Owens, Anna Duncan, Leah Carreon

**Affiliations:** 1 Orthopedic Surgery, University of Louisville School of Medicine, Louisville, USA; 2 Orthopedics, Norton Healthcare, Norton Leatherman Spine Center, Louisville, USA; 3 Orthopedic Surgery, University of Louisville, Louisville, USA; 4 Research, Norton Healthcare, Norton Leatherman Spine Center, Louisville, USA

**Keywords:** abm/p-15, complications, fusion, outcomes, recombinant human bone morphogenetic protein-2, rhbmp-2, tlif, transforaminal lumbar interbody fusion

## Abstract

Objective: This retrospective observational cohort study evaluated the efficacy and safety of anorganic bone matrix combined with a P-15 osteogenic cell-binding peptide (ABM/P-15) in one- to two-level transforaminal lumbar interbody fusion (TLIF), compared with recombinant human bone morphogenetic protein-2 (rhBMP-2) or local autograft bone.

Background: ABM/P-15 is a relatively new peptide-based bone graft substitute that promotes new bone formation. While its safety and efficacy have been demonstrated in randomized trials of anterior cervical fusion, evidence supporting its use in lumbar fusion remains limited, and clinical outcomes have not been well described.

Methods: We retrospectively reviewed 318 patients who underwent one- or two-level TLIF. Patients lacking complete patient-reported outcome measures (PROMs) or 12-month radiographic evaluations were excluded. Patients were categorized into three groups based on graft material: ABM/P-15, rhBMP-2, and local autograft bone. Each ABM/P-15 patient was matched to a rhBMP-2 and local autograft patient based on demographics, number of levels fused, and graft dose/kit size. Postoperative complications, PROMs, and fusion rates were compared among the groups.

Results: Each group consisted of 38 patients after matching. The mean ABM/P-15 volume was 4.4 ± 1.3 cc (cost: $2,432.3 ± 714.4), and the mean rhBMP-2 dose was 3.0 ± 1.1 mg (cost: $2,316.6 ± 843.0). At 12 months, PROMs improved significantly in all groups, with no significant differences among them. Fusion was achieved in 84% of the ABM/P-15 group, 79% in the rhBMP-2 group, and 82% in the autograft group (p = 0.265). Complication rates, including postoperative radiculopathy, were also comparable (p = 0.725).

Conclusion: To the best of our knowledge, this is the first study to directly compare ABM/P-15 and rhBMP-2 in patients undergoing TLIF. ABM/P-15 demonstrated comparable fusion and complication rates to rhBMP-2 in one- or two-level TLIF procedures, at a similar cost.

## Introduction

Transforaminal lumbar interbody fusion (TLIF) has been widely performed since being introduced in 1982 [1]. The primary goal of TLIF is to stabilize the spine by achieving arthrodesis through an interbody cage packed with bone graft. Local autograft bone harvested during laminectomy and facetectomy is widely used and has demonstrated reliable fusion rates [2]. However, local bone becomes less effective in multilevel fusions or difficult fusion environments such as smoking, poor bone quality, and fusion at the L5/S1 level [3-5]. In this case, autologous iliac crest bone graft (ICBG) has traditionally been used; however, it is now often avoided due to the associated risks of complications and the additional operative time and dissection required for graft harvesting [6,7].

Various alternative bone grafts have been adopted to enhance fusion, including demineralized bone matrix (DBM), synthetic bone grafts such as ceramics composed of hydroxyapatite and calcium phosphate, and bioactive molecules such as bone morphogenetic protein (BMP) [8,9]. In lumbar fusion surgery, recombinant human bone morphogenetic protein-2 (rhBMP-2; INFUSE, Medtronic, Minneapolis, MN, USA) is potentially the current gold standard for supplemental bone grafting, as it eliminates the risk of infectious disease transmission and possesses osteoinductive properties [10-12]. Although unique complications have been reported with rhBMP-2, including postoperative radiculitis, inflammatory responses, and potential malignancy, large studies and meta-analyses have demonstrated that rhBMP-2 enhances bony fusion without significantly increasing the risk of these complications [13-15]. However, its high cost remains a concern, contributing to the overall healthcare economic burden [16].

Anorganic bone matrix combined with a P-15 osteogenic cell-binding peptide (ABM/P-15; i-Factor, Cerapedics, Inc., Broomfield, CO, USA) is a relatively new peptide-based graft substitute, classified as a bioactive molecule in the same category as BMP. It enhances new bone formation by promoting cell attachment to collagen fibers and improving cell viability [17,18]. Approved for anterior cervical fusion surgery in 2015, randomized trials have since demonstrated its safety and efficacy in anterior cervical procedures [19,20]. However, research on its use in lumbar fusion remains limited, and its clinical outcomes have not been well described. This study aimed to assess the efficacy, safety, and cost of ABM/P-15 in TLIF, compared with rhBMP-2 or local autograft bone with no bioactive molecules.

## Materials and methods

We retrospectively reviewed clinical records of a consecutive series of patients who underwent one- or two-level TLIF with an interbody cage for lumbar disc herniation, mechanical disc collapse, or degenerative spondylolisthesis at a single institution between January 2013 and December 2021. Standard demographic and preoperative risk stratification data were collected, including age, sex, body mass index (BMI), smoking status, diabetes mellitus, and American Society of Anesthesiologists (ASA) grade [21]. Patient-reported outcome measures (PROMs), including the Oswestry Disability Index (ODI) and Numeric Rating Scale (NRS, 0 to 10) for back and leg pain, were recorded preoperatively and at one year postoperatively [22-24]. Surgical parameters collected included fusion levels, operative time, and estimated blood loss (EBL). Hospital length of stay (LOS) was measured in hours.

Patients were categorized into three groups based on the bone graft used: ABM/P-15, rhBMP-2 (BMP), and local autograft bone without the use of bioactive molecules (autograft). Both ABM/P-15 and rhBMP-2 were used in combination with local autograft. The choice of bone graft was based on the surgeon's preference. The volume and dose of ABM/P-15 and rhBMP-2 were recorded. ABM/P-15 was available in 1cc, 2.5cc, and 5cc kits. In order to be able to compare costs, only patients with rhBMP-2 kits that were xx-small (1.05 mg), x-small (2.1 mg), and small sizes (4.2 mg) were included in the study. Costs incurred by the hospital for these bone graft substitutes were provided by a senior financial analyst.

The primary outcome was postoperative complication rates. Secondary outcome included PROMs, fusion rates, and cost. Postoperative complications were defined as any adverse event requiring intervention within 90 days of surgery. Bony fusion was assessed at the one-year follow-up using X-ray or computed tomography (CT) as indicated on the radiology reports and defined as the presence of bridging bone formation between the upper and lower vertebral bodies or the formation of a thick fusion mass around the cages. Patients with incomplete PROMs data or missing radiographic evaluations at 12 months were excluded.

Statistical analysis

To control for bias inherent in a retrospective study, ABM/P-15 patients were propensity-matched to BMP and autograft patients based on age, sex, smoking status, BMI, ASA grade, insurance status, number of levels fused, and dose/kit size (1cc: xx-small, 2.5cc: x-small, and 5cc: small) [25]. A propensity score, the probability of a subject being in the ABM/P-15 cohort, was determined using binary logistic regression. Each patient in the ABM/P-15 group was matched to a rhBMP-2 and local autograft patient with the closest propensity score using nearest neighbor matching using a caliper width of 0.2 standard deviations (SDs) of the logit of the propensity score [26]. The postoperative complication rates, PROMs, fusion rates, and cost were compared among the groups.

Continuous variables were summarized as the mean ± SD, and categorical variables as frequencies with percentages. The Shapiro-Wilk test was used to assess the normality of continuous variables. Student’s t-test was used to compare groups for normally distributed continuous variables, and the Mann-Whitney U test was applied for non-normally distributed continuous variables. Categorical variables were compared using Fisher’s exact test. Analysis of variance (ANOVA) was used for comparisons among three groups. Statistical significance was defined as p < 0.05. All analyses were performed using IBM SPSS Statistics for Windows, Version 29 (Released 2022; IBM Corp., Armonk, New York, United States).

## Results

The primary study cohort included 67 patients with ABM/P-15, 133 with BMP, and 118 with local autograft (Table 1).

**Table 1:** Comparison of demographics and surgical parameters among three groups - patients treated with anorganic bone matrix combined with a P-15 osteogenic cell-binding peptide (ABM/P-15), recombinant human bone morphogenetic protein-2 (rhBMP-2), and autograft without bioactive molecules - in the original cohort. BMP: bone morphogenetic protein; SD: standard deviation

Parameter	ABM/P-15	BMP	Autograft	p-value
Number of patients	67	133	118	-
Age, years, mean (SD)	56.64 (13.38)	61.9 (11.87)	56.34 (12.31)	<0.001
Body mass index, kg/m^2^, mean (SD)	31.86 (5.73)	31.4 (6.57)	32.61 (6.46)	0.318
Males, N (%)	32 (48)	52 (44)	56 (42)	0.749
American Society of Anesthesiologists (ASA), N (%)				
1	1 (1)	1 (1)	2 (2)	0.843
2	30 (45)	40 (34)	51 (38)	
3	35 (52)	63 (53)	78 (59)	
4	1 (1)	4 (3)	2 (2)	
Smoker, N (%)	7 (1)	6 (5)	24 (18)	0.103
Diabetic, N (%)	7 (1)	24 (2)	29 (22)	0.134
Pre-operative, N (%)	7.39 (9.11)	6.73 (2.23)	6.53 (2.29)	0.543
Back pain, N (%)	5.71 (3)	6.75 (2.38)	6.41 (2.51)	0.081
Leg pain, N (%)	48.54 (15.78)	52.8 (15.45)	49.54 (18.35)	0.309
Oswestry Disability Index, N (%)	48.5 (15.8)	52.8 (15.5)	49.5 (18.4)	0.309
Number of levels, N (%)				
1	48 (72)	71 (53)	104 (78)	0.005
2	17 (25)	36 (31)	28 (21)	
3	2 (3)	5 (4)	1 (1)	
4	-	6 (5)	-	
Estimated blood loss, mL, mean (SD)	339.39 (230.05)	346.38 (271.59)	292.08 (172.61)	0.152
Operative time, mins, mean (SD)	278.13 (56.23)	294.92 (75.56)	209.4 (60.12)	<0.001
Length of stay, hours, mean (SD)	74.82 (47.32)	81.11 (52.64)	72.8 (42.85)	0.373

After propensity score matching, each group consisted of 38 patients, with no significant differences in demographic or surgical parameters among the groups, except for operative time (ABM/P-15: 245.6 ± 63.4 minutes, BMP: 272.8 ± 48.3 minutes, BMP: 245.6 ± 63.4 minutes, autograft: 210.4 ± 63.0 minutes, p < 0.001) (Table 2).

**Table 2:** Comparison of demographics and surgical parameters among three groups - patients treated with anorganic bone matrix combined with a P-15 osteogenic cell-binding peptide (ABM/P-15), recombinant human bone morphogenetic protein-2 (rhBMP-2), and autograft without bioactive molecules - in the original cohort following 1:1 propensity score matching. BMP: bone morphogenetic protein; SD: standard deviation

Parameter	ABM/P-15	BMP	Autograft	p-value
Number of patients	38	38	38	-
Age, years, mean (SD)	62.3 (12.1)	56.8 (12.72)	57.9 (11.4)	0.087
Body mass index, kg/m^2^, mean (SD)	32.4 (6.7)	31.5 (5.2)	32.8 (6.5)	0.576
Males, N (%)	17 (45)	17 (45)	15 (39)	0.773
American Society of Anesthesiologists (ASA), N (%)				
2	15 (39)	21 (55)	15 (39)	0.642
3	22 (58)	16 (42)	22 (58)	
4	1 (3)	1 (3)	1 (3)	
Smoker, N (%)	5 (13)	4 (11)	4 (11)	0.801
Diabetic, N (%)	15 (39)	16 (42)	14 (37)	0.107
Pre-operative, N (%)				
Back pain, N (%)	6.71 (2.25)	6.32 (2.27)	6.6 (2.3)	0.750
Leg pain, N (%)	6.8 (2.2)	5.58 (2.79)	6.5 (2.5)	0.081
Oswestry Disability Index, N (%)	54.9 (14.8)	49.3 (15.2)	49.0 (18.8)	0.187
Number of levels, interbody fusion, N (%)				
1	34 (89)	37 (97)	35 (92)	0.1
2	4 (11)	1 (3)	3 (8)	
Estimated blood loss, mL, mean (SD)	269.1 (184.6)	320.8 (216.2)	301.8 (180.9)	0.480
Operative time, mins, mean (SD)	245.6 (63.4)	272.8 (48.3)	210.4 (63.0)	-
Length of stay, hours, mean (SD)	63.6 (47.1)	75.0 (37.3)	77.5 (45.00)	0.253

The mean volume of the ABM/P-15 used was 4.4 ± 1.3 cc, with 8% using the 1 cc kit, 13% using the 2.5 cc kit, and 79% using the 5.5 cc kit. The mean dose of rhBMP-2 was 3.0 ± 1.1 mg, with 3% using the xx-small kit, 55% using the x-small kit, and 42% using the small kit. The mean cost was $2,432.3 ± 714.4 for ABM/P-15 and $2,316.6 ± 843.0 for rhBMP-2 (Table 3).

**Table 3:** Comparison of the dose and cost of bone graft materials between the two groups - ABM/P-15 and rhBMP-2. ABM/P-15: anorganic bone matrix combined with a P-15 osteogenic cell-binding peptide; rhBMP: recombinant human bone morphogenetic protein-2; BMP: bone morphogenetic protein; SD: standard deviation

Parameter	ABM/P-15	BMP
rhBMP dose, mg, mean (SD)	-	3.0 (1.1)
ABM/P-15 volume, cc, mean (SD)	4.4 (1.3)	-
Volume/kit size, N (%)		
1cc/xx-small kit	3 (8)	1 (3)
2.5cc/x-small kit	5 (13)	21 (55)
5.0cc/small kit	30 (79)	16 (42)
Cost, mean (SD)	2432.3 (714.4)	2316.6 (843.0)

PROMs improved at 12 months postoperatively, with no significant differences observed among the groups (Table 4).

**Table 4:** Comparison of patient-reported outcome measures and complication rates among three groups - patients treated with anorganic bone matrix combined with a P-15 osteogenic cell-binding peptide (ABM/P-15), recombinant human bone morphogenetic protein-2 (rhBMP-2), and autograft without bioactive molecules - in the original cohort following 1:1 propensity score matching. BMP: bone morphogenetic protein; POCD: postoperative cognitive dysfunction; AKI: acute kidney injury; DVT: deep vein thrombosis; SD: standard deviation

Parameter	ABM/P-15	BMP	Autograft	p-value
12-month post-operative, mean (SD)				
Back pain	4.1 (3.1)	4.2 (3.1)	3.6 (2.9)	0.649
Leg pain	4.3 (3.2)	3.9 (3.2)	3.5 (3.2)	0.479
Oswestry Disability Index	36.4 (21.5)	35.7 (24.0)	32.1 (23.6)	0.584
Fusion status, N (%)				
Nonunion	4 (11)	7 (18)	3 (8)	0.265
Partial	2 (5)	1 (3)	4 (11)	
Solid	32 (84)	30 (79)	31 (82)	
Complications, N (%)				
Radiculopathy	1 (3)	3 (8)	2 (5)	0.725
Dural tear	4 (11)	4 (11)	2 (5)	0.656
Urinary retention	2 (5)	1 (3)	1 (3)	0.847
Ileus	6 (16)	1 (3)	2 (5)	0.086
POCD	1 (3)	2 (5)	0	0.327
AKI	2 (5)	1 (3)	2 (5)	0.744
DVT	0	1 (3)	1 (3)	0.552
Cage reposition	0	1 (3)	1 (3)	0.552
Wound infection/epidural hematoma	2 (5)	3 (8)	2 (5)	0.797

Solid fusion was achieved in 84% of the ABM/P-15 group, 79% of the BMP group, and 82% of the autograft group. Obvious nonunion was observed in 11%, 18%, and 8% of these groups, respectively, with no statistically significant differences (p = 0.265). Complication rates also did not significantly differ among the groups, including postoperative radiculopathy (ABM/P-15: 3%, BMP: 8%, autograft: 5%, p = 0.725).

## Discussion

To the best of our knowledge, this is the first study to directly compare two bioactive molecular bone grafts, ABM/P15 and rhBMP2, in patients undergoing TLIF. Our findings showed that ABM/P-15 was comparable to rhBMP-2 in terms of fusion rates, complication rates, and cost in one- or two-level TLIF cases.

Five published studies evaluating the efficacy of ABM/P-15 in lumbar fusion surgery have reported its positive effects in promoting bony fusion without increased risk of complications. Mobbs et al. reported that the use of ABM/P-15 facilitated successful fusion and improved clinical outcomes in patients undergoing anterior interbody fusion [27]. Downie et al., in their randomized trial of 101 patients, reported that those undergoing lumbar non-instrumented posterolateral fusion with ABM/P-15 had significantly higher fusion rates compared to allograft bone graft mixed with locally harvested bone, at one-year (50% vs. 20%, p < 0.001) and five-year follow-up (57% vs. 30%, p = 0.037) [28,29]. Similarly, Lauweryns and Raskin, in their prospective study of 40 patients who underwent posterior lumbar interbody fusion (PLIF), compared ABM/P-15 and autograft by placing two cages within the same disc space - one filled with ABM/P-15 and the other with autograft - and found that ABM/P-15 significantly promoted early bony fusion, achieving 97.7% fusion at six months compared to 59.9% with autograft [30]. Sathe et al. conducted a direct comparison between ABM/P-15 and other alternative graft materials. In their retrospective review of 140 patients who underwent lateral and anterior interbody fusion, they found that bony fusion occurred significantly earlier in the patients with ABM/P-15, with a mean time to union of four months for ABM/P-15, compared to 10 months for rhBMP-2 and nine months for DBM [31]. All of them reported no increased risk of complications associated with ABM/P-15.

Consistent with previous reports, our study showed that no increased risk of complications was observed in the ABM/P-15 group. Although the difference was not statistically significant, the differing biological mechanisms between ABM/P-15 and rhBMP-2 may contribute to the reduced risk of postoperative radiculopathy by using ABM/P-15 instead of rhBMP-2 [32,33]. The rhBMP-2 is known for its strong osteoinductive properties, whereas ABM/P-15 is primarily osteoconductive [34]. Although the exact pathophysiology of rhBMP-2-associated radiculopathy remains unclear, it is believed to result from inflammation and/or ectopic bone formation near the nerve root, potentially triggered by the inflammatory response induced by rhBMP-2 [35,36]. In contrast, ABM/P-15 promotes cellular viability within the inorganic bone matrix without provoking an immunologic reaction [37].

We also found no significant differences in fusion rates among the patients with ABM/P-15, rhBMP-2, and those with only local allograft following one- or two-level TLIF. While the choice of graft material may be less critical than surgical factors such as adequate disc preparation in one- or two-level TLIF [33,38], one possible explanation includes the quality of autograft bone, which was not assessed in this study. Since both ABM/P-15 and rhBMP-2 were used in combination with locally harvested bone graft in this study, fusion outcomes may be negatively affected when local bone lacks sufficient osteogenic or osteoinductive properties. Although we attempted to minimize confounding through propensity score matching, there may be selection bias in graft choice during surgery. Surgeons may have preferred to use ABM/P-15 or rhBMP-2 in patients with poor bone quality, while relying on local bone alone in those with good quality.

It should be noted that these findings may vary depending on the amount of bone graft alternative used [33,39-41]. Although higher fusion rates have been reported with larger BMP doses, the BMP dose included in this study was low to match the cost of ABM/P-15. This may have resulted in the relatively low fusion rate in the BMP group and no difference in fusion rates compared to the autograft group. Moreover, as the dose-effectiveness of ABM/P-15 remains unclear, the optimal dose per level for TLIF may be higher or lower than what was used in our cohort. Differences in effectiveness between ABM/P-15 and BMP may be observed in more challenging cases or in multilevel fusions.

This study has several limitations. First, it was a retrospective analysis conducted at a single institution with several surgeons who may have differences in surgical technique and graft choice, dosing, and placement. Second, the sample size was relatively small. Third, the assessment of fusion was based on radiology reports. Lastly, the type and dose of graft materials were not standardized, based on surgeon preference, and as mentioned above, the potential for selection bias cannot be excluded. Further studies are needed to evaluate optimal dosing as well as the efficacy and safety of ABM/P-15.

## Conclusions

This study directly compares ABM/P-15 and rhBMP-2 in patients undergoing TLIF. Our findings suggest that ABM/P-15 may be comparable to rhBMP-2 in terms of fusion rates and complication rates in one- or two-level TLIF, at similar costs in this small single-center cohort. Further prospective, multicenter, and dose-optimized studies are warranted to confirm these findings and to determine the ideal indications and graft volumes for ABM/P-15 in more complex fusion environments.
